# Reflective Optical Chopper Used in NIST High-Power Laser Measurements

**DOI:** 10.6028/jres.113.024

**Published:** 2008-12-01

**Authors:** Xiaoyu Li, Thomas Scott, Chris Cromer, Joshua Hadler

**Affiliations:** National Institute of Standards and Technology, Boulder, CO 80305

**Keywords:** attenuator, beamsplitter, calorimeter, device under test, high-power laser, infrared (IR), laser detector calibration, laser power meter, monitor detector, reflective optical chopper, wedge-shaped transparent material, visible aiming laser, ZnSe

## Abstract

For the past ten years, NIST has used high-reflectivity, optical choppers as beamsplitters and attenuators when calibrating the absolute responsivity and response linearity of detectors used with high-power CW lasers. The chopper-based technique has several advantages over the use of wedge-shaped transparent materials (usually crystals) often used as beam splitters in this type of measurement system. We describe the design, operation and calibration of these choppers. A comparison between choppers and transparent wedge beampslitters is also discussed.

## 1. Introduction

High-power, continuous-wave (CW), infrared (IR)-emitting lasers are commonly used in industry to cut, weld, and process various types of materials. Although laser power measurements have been performed at NIST for more than twenty-five years, the growth of new industrial laser-based systems over the past ten years has increased the need for measurements at higher powers over a wider wavelength range than previously required. Industrial users of high-power CW carbon dioxide (CO_2_) lasers emitting radiation at a wavelength of 10.6 μm were the first customers for NIST detector calibration services. To accommodate this need, scientists at NIST designed and built the K-series calorimeters [1] to handle laser powers from 5 W to 1000 W. Later, high-power CW neodymium-doped yttrium aluminum garnet (N_d_:YAG) lasers emitting radiation at 1.06 μm became increasingly popular and, consequently, NIST added an appropriate calibration service for these laser users about ten years ago. Today, both diode and fiber lasers (emitting IR radiation from 800 nm to 2 μm) are widely used by high-power industrial laser users. We expect that a NIST detector calibration service at these wavelengths will also be required in the near future.

In recent years we have used spinning, reflective optical choppers (ROCs) as beamsplitters in the NIST high-power, detector calibration system, instead of wedge-shaped transparent materials (WTMs), which had been used for this purpose over the past 25 years. This method has advantages over the use of WTMs, such as ease of use, enhanced safety for the user, and increased measurement-system flexibility. For these same reasons, we also employ ROCs as beam attenuators in the measurement system [2] for characterizing the response linearity of detectors used with high-power lasers.

## 2. NIST High-Power Calibration System

**Fig. 1:**
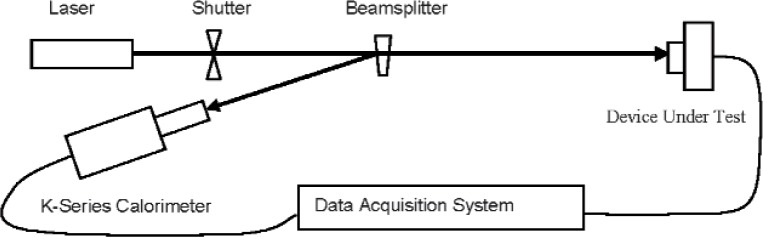
Previous NIST Calibration System with wedge beamsplitter.

Figure 1 shows a diagram of the previous NIST high-power laser detector calibration system in which a WTM was used as a beamsplitter to provide simultaneous beams to the device under test (DUT) and the primary standard calorimeter. In order to obtain the desired power-splitting ratio of the transmitted to the reflected beams, materials with the appropriate index of refraction at the operating laser wavelength were used. WTM also had to have good thermal stability and low absorptivity, since the measurements are performed using a high-power laser. For most calibration measurements at 10.6 μm, the system used an uncoated ZnSe wedge (2°) beamsplitter along with two primary standard K-series calorimeters serving as reference standards. The beamsplitter was aligned (relative to the incident laser beam) such that a small angle of incidence (< 5°) was produced in order to minimize the influence of any polarization changes during the measurement process. In addition, long beam paths were used so that the unused beams from the beamsplitter could be easily blocked or shielded from the detectors. The beamsplitter ratio value (that is, the ratio of transmitted to reflected power) was measured by two NIST K-series calorimeters. An accurate value of the beam-splitter ratio was calculated from two sets of ratio data obtained by exchanging the positions of these calorimeters. Since the beamsplitter ratio measurement was a time consuming task typically taking 7–8 hours to complete, it was not practical to measure the beamsplitter ratio each time a calibration was performed, contributing to a larger overall measurement uncertainty. Calibrations were then performed by comparing the DUT to one of the K-series calorimeters using the previously measured beamsplitter ratio value [3].

In the mid-1990’s, we began using lasers with higher divergences, for example, high-power flash-lamp pumped Nd:YAG lasers. Shorter beam paths were used, in order to be adapted for the higher-diverged laser beam; thus, making it very difficult to block the unused, higher order beams coming from the beamsplitter. At that time we began testing the use of ROCs in this measurement system since they produce only two beams. This technique was found to work well experimentally and, subsequently, we began using ROCs for certain detector calibration measurements.

**Fig. 2:**
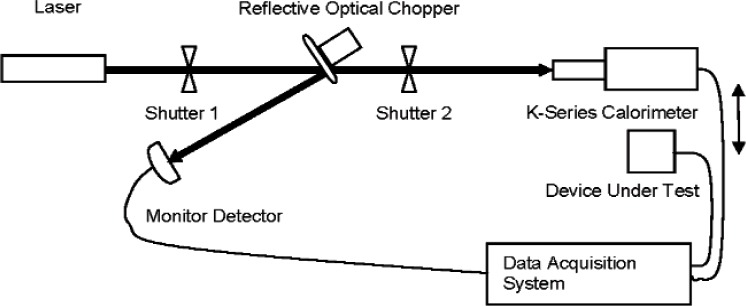
Current NIST Calibration System using a ROC and Monitor Detector.

Several years ago, we re-designed our calibration system to incorporate a monitor detector and second shutter combination [4], at which time the ROC technique became the only beamsplitter technique used for both 1.06 μm and 10.6 μm high-power measurements. Figure 2 shows a diagram of the current NIST high-power laser detector calibration system. The detector calibration is performed in three steps: (1) relative ROC ratio measurements in which a standard calorimeter is placed in one beam and a monitor detector is placed in the other beam and then the two detector outputs are compared; (2) the standard calorimeter is replaced by the DUT to be calibrated and the two detector outputs are compared; then (3) the first step is repeated. The calibration result is calculated from the three sets of data. This technique is based on the assumption that the surface reflectivity of the ROC remains constant during the entire calibration process (but knowledge of the absolute power-splitting ratio is no longer required).

## 3. Design, Operation, and Calibration of the ROC

**Fig. 3:**
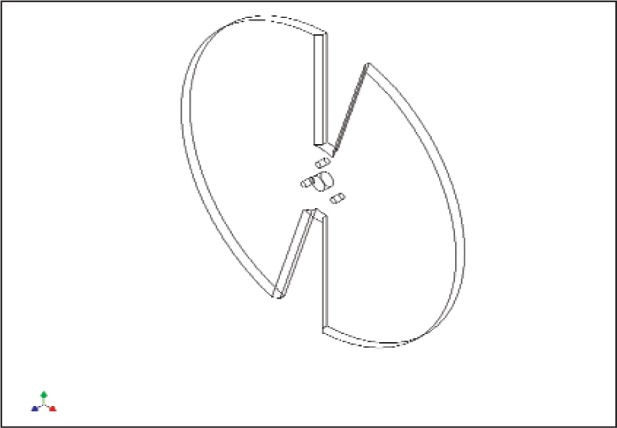
Typical Reflective Optical Chopper.

Figure 3 shows the typical ROC design now in use, which is a 20.32 cm in diameter, 0.64 cm thick, aluminum disc that has several, equally spaced, radially-oriented, open sectors (between the blades) precisely cut by an EDM (Electrical Discharge Machine). Radially-oriented sectors help assure that the ratio of power transmitted to that reflected is constant at all radial positions of the beam. To avoid clipping the beam, when the incident angle of the laser beam is not zero, 45 beveled edges were machined on the back surface of each blade along all the openings. The disc surfaces were first black anodized and then the front surface was diamond-turned to produce a highly reflective surface to minimize the amount of absorbed radiation. A fresh diamond-turned aluminum surface has very similar reflectivity to vacuum-deposited aluminum (greater than 95 % at 1.06 μm) [5]. It’s important to choose the proper material for constructing the ROC to minimize the grating effect (i.e., multiple reflected beams) from the grooves produced on the diamond-turned surface at the laser wavelength to be used. For example, aluminum alloy 6061 (T6) was found to work well for 1.06 μm. The reflectivity of the diamond-turned aluminum surface will decrease with time, but this degradation process has been found to be very slow. For example, the reflectivity of one of our ROCs decreased only 2 % over a ten-year period.

The ROC is mounted on an electric motor assembly consisting of an electrical motor (rotation rate of 1500 rpm) and 2:1 belt driving system. This assembly serves two purposes: (1) lowers the rotating speed of the ROC to 750 rpm to reduce the effect of air turbulence on the rest of the calibration system, and (2) reduces the diameter of the ROC mounting axis, thus, allowing a larger useable area for a given diameter of the chopper. During operation, the entire assembly is placed on a magnetic base or a moving translation stage depending on the application.

The ROC can be used as either a beamsplitter or an attenuator in the calibration system. When the ROC is used as a beamsplitter, both transmitted and reflected beams are used for the measurement, whereas, when the ROC is used as an attenuator, only the transmitted beam is used (the reflected beam is blocked). The attenuation value of the ROC is determined by its geometry (that is, the ratio of open to active surface area of the sectors) while the surface reflectivity has no effect on this value. We have used both mechanical and optical methods to determine the attenuation value of the ROC. The mechanical method was performed using a precision optical comparator to determine the exact angle of the radially-oriented openings of the sectors. This information was then used to calculate the ratio of surface area to open area of the sectors. The optical method was performed using the NIST C-series calibration system [6] to measure the attenuation value directly. The results from two methods agreed within 0.1 %, but only the attenuation value measured by the optical method was used in our calibration process.

## 4. Thermal Stability of the ROC

The stability of the geometrical shape of the ROC during the measurement process is critical for both beamsplitter and attenuator functions; thus, knowledge of the expected temperature rise and thermal expansion properties of the material is important. The thermal expansion coefficient of aluminum alloys is typically 2.5 × 10^−5^/°C [7]. To investigate this effect, we measured the temperature rise of an ROC (which had a nominal 50/50 splitter ratio) exposed to 1000 W from a CO_2_ laser. The temperature of the ROC rose 6 °C in the first 60 seconds and remained at a stable temperature until our final measurement at 6 minutes. The relatively low temperature rise of the ROC is due to rapid heat diffusion throughout the metallic disk, in addition to the self-cooling action produced by its rapid rotation in the air. The normal exposure time of the ROC when used in the NIST high-power calibration system varies from 10 to 300 seconds.

## 5. Dual ROC Application

For detector absolute calibration or response linearity measurements with high laser powers, the NIST measurement system employs two ROCs. One ROC serves as a beamsplitter and directs the reflected beam to the monitor detector to record laser power fluctuations during the measurement process. The other ROC is positioned in front of the K-series calorimeter (absolute calibration) or the DUT (response linearity measurement) and serves as an attenuator for the incident radiation. Since the ROCs effectively “chop” the CW laser beam, it’s possible to get “interference” between the two ROCs, thus, causing an error in the calibrated attenuation value of the attenuator-ROC. This effect was eliminated by having (1) the blade of the beamsplitter-ROC narrower than the blade of the attenuator-ROC, (2) center lines of both blades passing through the laser beam simultaneously (to accomplish this phase synchronicity, we designed and built two ROC phase detection devices, which are used in conjunction with two, computer-programmed, encoded DC motors), and (3) data correction by the attenuation value of the beamsplitter-ROC.

## 6. Advantages of ROC Over WTM

The following are the main desirable properties for ROCs compared to WTMs in the NIST high-power measurement system:

1. Safety: Unlike the uncoated optical wedge beamsplitter, the ROC has only one reflected beam and one transmitted beam; consequently, no extra, high-order beams need to be blocked. Naturally, this is a much safer situation for operators of the high-power laser system operation.
2. Alignment: At the present time, the majority of the NIST high-power lasers emit radiation in the IR wavelength region, so a visible aiming laser (VAL) is usually employed to align the various optical components and detectors of the measurement system. Because the refractive index of the WTM is wavelength dependent, the directions of the transmitted beams will be different for the high-power IR laser and VAL. In contrast, the ROC is wavelength independent, which allows more accurate beam alignment.
3. Flexibility and stability: The beamsplitter ratios of uncoated, WTM beamsplitters are determined by their indexes of refraction and are, consequently, fixed (and limited) for each laser wavelength used and type of material available. However, since ROC ratios and attenuator values are determined by their geometries alone (for a given surface reflectance), they can be easily machined to achieve a wide range of splitter (or attenuator) values. Although coatings can be used on the WTM to achieve various splitter and attenuator ratios, we generally avoid these due to such potential problems as thermal stability, sensitivity to angular alignment, wavelength dependence, and damage susceptibility when cleaned. In addition, thermal stability is a substantial issue when using high-power lasers since the radiation absorbed by the splitter is converted to heat, and is typically difficult to remove from transparent optical materials.
4. Calibration: The attenuation value of an ROC can be measured at any laser wavelength and then be used for all laser wavelengths. This allows calibrations to be performed once only using the NIST laser calibration system having the highest accuracy. For example, low power visible-laser measurements can generally be performed with much higher accuracy than high-power IR measurements.

## 7. Discussions

Despite all the advantages of ROCs, they can only be used with CW lasers and slow (long time constant) detectors since they optically “chop” the laser beam. For instance, the “chopping” frequency will be 50 Hz for typical ROCs with four blades; therefore, they are not suitable for Q-switched lasers and photon detectors. Fortunately, most high-power industrial lasers are either CW or quasi-CW (that is, pulsed at high repetition rates) and most detectors in use are relatively-massive thermal detectors with long time constants.

## 8. Conclusions

After approximately ten years of use with various lasers in our calibration laboratory, we have found ROCs to work well for calibrating detectors used with high-power IR lasers. We have found them to be stable and reliable over time and, as a result, have helped to improve our high-power laser detector calibration system.
